# Multifunctional System with Ferulic Acid: Increased Safety, Antioxidant Activity, and Efficacy of Sunscreen Formulations

**DOI:** 10.3390/antiox15070885

**Published:** 2026-07-17

**Authors:** Jamile Vitória Alves Freire, Fernanda Ílary Costa Duarte, Lívia Maria da Costa Dantas, Júlia Luíza André Santana, Patrícia Prado Augusto, Cristiane Fernandes de Assis, Francisco Caninde de Sousa Junior, Ana Paula Barreto Gomes, Fernando Henrique Andrade Nogueira, Patricia Santos Lopes, André Rolim Baby, Ádley Antonini Neves de Lima, Elissa Arantes Ostrosky

**Affiliations:** 1 Pharmaceutical Sciences Postgraduate Program, Center of Health Sciences, Federal University of Rio Grande do Norte, Natal 59012-570, Brazil; 2 Hospital Pharmacy, Hospital João Machado, Natal 59012-570, Brazil; 3 Department of Pharmaceutical Sciences, Federal University of São Paulo, São Paulo 09913-030, Brazil; 4 Department of Pharmacy, Center of Health Sciences, Federal University of Rio Grande do Norte, Natal 59012-570, Brazil; 5 Department of Pharmacy, Faculty of Pharmaceutical Sciences, University of São Paulo, São Paulo 05508-000, Brazil

**Keywords:** ferulic acid, multicomponent system, synthetic antioxidants, cosmetic, sun protection factor, skin release

## Abstract

The topical application of ferulic acid (FA) is limited by its low solubility and high susceptibility to oxidation. To overcome these limitations, the multicomponent system Cicloferulic^®^ (CF) was developed by mixing FA, hydroxypropyl β-cyclodextrin (HPβ-CD), and polyvinylpyrrolidone K30 (PVP K30), which demonstrated greater safety and antioxidant efficacy than FA alone. The physicochemical characterization of FA, CF, and their components showed greater thermal stability and reduced crystallinity of CF, indicating the formation of the system. In cytotoxicity assays on HaCaT cells, FA maintained cell viability only up to 500 µg/mL, while CF maintained viability even at 10.000 µg/mL, demonstrating 20 times greater safety than FA. In cellular antioxidant activity assays, FA and CF showed similar effects at low concentrations. In the 1,1-diphenyl-2-picrylhydrazyl (DPPH) method, CF and FA reduced more than 70% of DPPH; however, CF contains only 12% FA, indicating an eightfold greater efficiency than FA. Formulations containing FA and CF increased the Sun Protection Factor (SPF) in vitro, even with a 50% reduction in UV filters. However, they did not stabilize formulations with intrinsically photolabile UV filters. In release studies, FA exhibited rapid flow, while CF promoted a modified release profile. Thus, CF stands out as an innovative, multifunctional system that can improve the stability, safety, and efficacy of FA for topical applications.

## 1. Introduction

With increased life expectancy, there is growing attention to studies of skin aging. Several studies have been conducted to develop new cosmetic products containing various active ingredients to combat and prevent the harmful effects of ultraviolet (UV) radiation, thereby reducing the incidence of skin cancer and delaying photoaging [1,2]. Strategies for preventing skin damage caused by UV radiation include not only the use of sunscreens, but also critical behavioral measures, such as avoiding direct exposure to the sun during periods of highest radiation intensity, reapplying sunscreen after sweating or bathing, and ensuring coverage of all exposed skin areas [3,4,5].

As for sunscreens, they can be broadly classified into chemical (organic) and physical (inorganic) types [5]. In summary, both organic and inorganic filters have multifactorial protection mechanisms that involve the absorption and dissipation of UV radiation energy, complemented by reflection and dispersion processes that enhance the photoprotective efficacy of cosmetic formulations [6,7,8]. Despite their high photoprotective efficacy, some of these substances have been associated with environmental impacts and skin hypersensitivity reactions, especially organic filters [9,10]. Thus, there is growing interest in the cosmetic market for ingredients that can minimize the effects of UV radiation, offering potentially safer alternatives. The combination of active molecules with traditional UV filters makes it possible to achieve higher or equivalent levels of sun protection (SPF) with lower concentrations of synthetic filters, reducing the risk of skin sensitization and environmental impacts, in addition to adding additional benefits to the formulation, since some of these molecules have antioxidant properties [10,11,12,13].

A class of active substances that has been increasingly explored are phenolic compounds, a broad group of secondary metabolites of plant origin characterized by the presence of one or more aromatic rings linked to hydroxyl groups, which have reducing properties due to their chemical structure, acting as free radical scavengers, transition metal chelators, both in the initial stages and in the propagation of the oxidative process, in addition to modulating inflammatory and enzymatic processes [14]. Ferulic acid (FA) is a phenolic compound found in various green leafy vegetables, with high concentrations in wheat bran, rice bran, and corn [15]. Its antioxidant mechanism of action primarily involves inhibiting the formation of reactive oxygen species (ROS), acting as a barrier at the cell membrane, and minimizing the effects of thymine dimers, carcinogenic agents resulting from skin exposure to UV radiation [16].

Due to its antioxidant activity, FA has been widely used in skin care formulations and also acts as an adjuvant in sunscreens [17]. The molecule has two isomeric forms, cis and trans. The trans isomer exhibits two characteristic absorption peaks at 307 nm and 284 nm, whereas the cis isomer shows a maximum around 316 nm, which favors its synergistic action with UV filters [15]. Its topical application inhibits erythema induced by ultraviolet (UV) radiation exposure, contributing to the reduction in the effects of skin photoaging and standing out as a promising compound for multifunctional photoprotective formulations [12,18,19]. However, its use is still limited by its low solubility in aqueous media and its tendency to rapid oxidation and decomposition into inactive products, which makes it potentially unstable [20].

New technologies have been used to address stability, bioavailability, and other physicochemical characteristics of pharmaceutical products. One alternative is the synthesis of multicomponent systems using cyclodextrins (CDs) and polymers [21]. CDs are cyclic oligosaccharides composed of glucose units linked by α-1,4 bonds, with a hydrophilic exterior and a hydrophobic interior, allowing the incorporation of various substances and contributing to better stability, solubility, and bioavailability [22]. Among cyclodextrin derivatives, hydroxypropyl-β-cyclodextrin (HPβ-CD) stands out for its aqueous solubility and amorphous character, which favor the formation of stable inclusion complexes that are less prone to precipitation and highly soluble [23,24,25]. In addition, the encapsulation of ferulic acid in the hydrophobic cavity of HPβ-CD may also protect against oxidative degradation [23,26].

The incorporation of water-soluble polymers into this system can increase complexation efficiency, enabling a significant reduction in the amount of CD required, thereby optimizing costs, reducing toxicity, and reducing the volume of the final product [27,28,29]. This technique can improve stability and antioxidant activity, allowing the use of smaller amounts of active ingredients to achieve the same effect [29,30].

The hydrophilic polymer polyvinylpyrrolidone K30 (PVP K30) exhibits co-complexing behavior, evidenced by its ability to increase the apparent stability constant and complexation efficiency of systems containing cyclodextrins, even at low concentrations [31]. This effect is related to the formation of ternary complexes stabilized by non-covalent interactions and hydrogen bonds, which favor greater solubility, improved dissolution performance, and inhibition of compound recrystallization compared to binary systems [25,32,33,34].

To increase the stability and solubility of ferulic acid and to assess its antioxidant and photoprotective potential, the multicomponent system Cicloferulic^®^ (CF) was developed, comprising FA, hydroxypropyl β-cyclodextrin (HPβ-CD), and the hydrophilic polymer polyvinylpyrrolidone K30 (PVP K30).

Given the technological potential of CF as a strategy to improve the stability, solubility, and performance of FA, we characterized CF and its components using spectroscopic, thermal, and structural analyses. We evaluated its safety and antioxidant activity in human keratinocyte cell lines (HaCaT). In addition, we assessed the in vitro skin release and photoprotective efficacy of cosmetic formulations containing FA and CF to examine how the multicomponent system influences FA behavior and performance. This integrated approach allows us to understand the potential of CF as a multifunctional cosmetic active ingredient.

## 2. Materials and Methods

### 2.1. Preparation of Cicloferulic^®^

FA and HPβ-CD were weighed in a 1:1 molar ratio, with the subsequent addition of PVP K30 polymer (*w*/*w*). After homogenization of the powders, an ethanol: water solution was added until a paste formed, which was then dried in an ECB 1.1 digital oven (Odontobrás, São Paulo, Brazil) at 50 °C. The resulting samples were stored in a desiccator and kept away from light [35].

### 2.2. Physical and Chemical Characterization

#### 2.2.1. Fourier Transform Infrared Spectroscopy (FTIR)

The isolated components and CF were analyzed by FTIR spectroscopy using an Attenuated Total Reflectance (ATR) accessory. The samples were placed directly on the ATR crystal of the Prestige-21 IR spectrometer (Shimadzu, Kyoto, Japan). The spectra were recorded over 700–4000 cm^−1^, with 20 scans and a spectral resolution of 4 cm^−1^.

#### 2.2.2. Thermal Analysis

##### Differential Scanning Calorimetry (DSC)

The DSC curves of the isolated components and CF were obtained using a DSC-60 (Shimadzu, Kyoto, Japan), using approximately 2 mg of each sample. The samples were placed in sealed aluminum crucibles and analyzed under a nitrogen atmosphere (100 mL/min) over a temperature range of 30 °C to 350 °C at a heating rate of 10 °C/min. The equipment was previously calibrated with an indium standard. The thermograms obtained were processed using Origin software (v8.0).

##### Thermogravimetry/Thermogravimetric Derivative (TG/DTG)

Thermogravimetric (TG) and derivative (DTG) analyses of the isolated components and CF were performed on a DTG thermobalance (Shimadzu, Kyoto, Japan), using approximately 5 mg of each sample. The analyses were conducted under a nitrogen atmosphere (130 mL/min), with a temperature range of 30 °C to 900 °C and a heating rate of 10 °C/min. The TG/DTG and DSC curves were interpreted using TA60WS software (Shimadzu, Kyoto, Japan).

#### 2.2.3. X-Ray Diffraction (XRD)

The analyses were performed using a high-resolution XRD-7000 X-ray diffractometer (Shimadzu, Kyoto, Japan) equipped with a Seifert ID3000 generator and copper radiation source (Cu Kα; λ = 1.5406 Å), angular range of 2θ between 3° and 70°, angular increment (step size) of 0.02°, and scanning speeds of 5°/min for rapid analyses and 1°/min for structural refinement analyses using the Rietveld method.

#### 2.2.4. Reduction in the Radical 2,2-Diphenyl-1-Picrylhydrazil (DPPH●)

Solutions of FA (12 mg/mL), PVP K30 (0.4 mg/mL), and HP-β-CD (17.4 mg/mL, maximum solubility) were prepared in absolute ethanol at the same proportions used to obtain the multicomponent system. CF was evaluated at concentrations of 0.5 mg/mL, 1 mg/mL, and 1.5 mg/mL. After treatment with DPPH● [36], the samples were evaluated at 495 nm using a UVM340 microplate reader (Biochrom Asys, Cambridge, UK). Antioxidant activity was expressed as a percentage of DPPH● inhibition calculated using the following equation:

(1) $$In\;vitro\;antioxidant\;activity\;(\%)=\;\left(\frac{Control\;absorbance-Sample\;absorbance}{Control\;absorbance}\right)\;\times \;100$$

The linear regression curve for calculating the CF concentration required to inhibit 50% of the DPPH radical (IC_50_) was constructed using concentrations ranging from 10 to 500 μg/mL, to determine the minimum concentration to incorporate into cosmetic formulations.

### 2.3. Cytotoxicity Assessment

The cytotoxicity assessment of CF, FA, CD, and PVP K30 was performed on an immortalized epithelial tissue cell line (Human Keratinocyte BBRJ Code nh-skp-KT0015) (HaCaT) cell line using the 3-(4,5-dimethylthiazol-2-yl)-2,5-diphenyltetrazolium bromide (MTT) reduction assay, used to determine cell viability [37,38]. The cells were cultured in supplemented Dulbecco’s Modified Eagle Medium (high glucose; Gibco) (DMEM), incubated at 37 °C with 5% CO_2_, and subjected to the assay until they reached approximately 80% subconfluence. The samples were prepared at predefined solvent concentrations, ensuring safe levels that did not affect cytotoxicity. All samples were applied to 96-well plates containing 1 × 10^4^ cells per well and incubated for 48 h.

Cell viability was assessed by converting MTT to formazan crystals. Absorbance was measured at 570 nm in a Synergy HT multidetection microplate reader (BioTek, Winooski, VT, USA), controlled by Gen5 software (v 1.11.5). The results were calculated using the following equation:

(2) $$MTT\;reduction\;(\%)=\frac{Treatment\;absorbance}{Control\;absorbance}\;\times 100$$

The cell viability curves were used to calculate IC_50_ and IC_10_ values via sigmoidal regression in Phototox^®^ software version 1.0 (2001).

### 2.4. Antioxidant Activity in Cells

The in vitro antioxidant activity of FA and CF was evaluated using an immortalized epithelial tissue cell line (Human Keratinocyte BBRJ Code nh-skp-KT0015) (HaCaT), cultured as described in the previous section (cytotoxicity assay), following the protocols of the Organization for Economic Cooperation and Development (OECD) and Good Laboratory Practice (GLP) guidelines [37,38]. The assays were performed in 96-well plates containing 5 × 10^4^ cells per well, with incubation for 24 h. Oxidative stress was induced with H_2_O_2_ (4 mol/L), and the dye 2′,7′-dichlorofluorescein diacetate (DCFH-DA, 2000 μM) was added to the cell medium.

Fluorescence intensity was measured using a Synergy HT multidetection microplate reader (BioTek, Winooski, VT, USA), equipped with UV-VIS and fluorescence detection, operated with Gen5 software, with excitation at 485 nm and emission at 538 nm. Resveratrol (250 mM) was used as a standard because of its well-established ability to neutralize reactive oxygen species [39,40]. In the presence of reactive oxygen species (ROS), DCFH is oxidized to the highly fluorescent dichlorofluorescein (DCF). Thus, the fluorescence intensity obtained was proportional to the amount of intracellular ROS [38,39].

The cells were treated with FA and CF at concentrations corresponding to IC_10_ and 100× IC_10_, to explore potential cytotoxic effects at extreme concentrations [41]. It is important to note that the highest concentration (100× IC_10_) does not reflect typical concentrations of topical exposure. The high concentration value is used to determine whether the activity is dose-dependent or whether the sample will act as a pro-oxidant.

### 2.5. Development of Photoprotective Formulations

For the development of photoprotective formulations, Butyl methoxydibenzoylmethane (UVA filter) and Ethylhexyl methoxycinnamate (UVB filter) filters were used in combination with FA and CF [42,43]. The concentration of CF was defined based on the calculation of IC50, while the concentration of FA corresponded to its proportion in that concentration of CF. The qualitative and quantitative composition (% *w*/*w*) of the formulations is described in Table 1.

#### In Vitro Photoprotective Efficacy and Photostability Test

The in vitro photoprotective efficacy was evaluated using the UV-2000S diffuse reflectance spectrophotometer (Labsphere, North Sutton, NH, USA) over the spectral range of 250 to 450 nm, with a 1 nm step. An amount of 1.3 mg/cm^2^ of each sample was uniformly applied to polymethyl methacrylate plates (Helioplate HD6, Helioscreen, North Sutton, NH, USA) [44].

After application, the plates were kept at room temperature, protected from light, for 30 min to dry [43,45]. The analyses were performed in triplicate, with measurements at nine different points per plate. The photoprotective efficacy was determined using the sun protection factor (SPF) and critical wavelength (λ_crit), expressed as mean ± standard deviation. The in vitro parameters were calculated using UV-2000 software (v 1.1.1.0), according to the methodology described by Cândido et al. [46].

The photostability test was adapted from Scalia and Mezzena, Cândido et al., and Pereira et al. [46,47,48]. The same substrates containing the samples were irradiated for up to 30 min in a solar simulator (Suntest CPS+, Atlas, Mount Prospect, IL, USA) equipped with a xenon lamp at 500 W/m^2^. After exposure to UV radiation, the post-irradiation SPF values and critical wavelength were determined again.

### 2.6. In Vitro Skin Release Study of Cosmetic Formulations

The skin release study was conducted in a Franz diffusion cell system with a diffusion area of 1.77 cm^2^ and a receiving-compartment volume of 7 mL. The cells were assembled by inserting the membrane between the donor and receiving compartments. Cellulose acetate filter membranes (VCWP04700, Millipore^®^, Darmstadt, Germany), previously impregnated with a lipid solution composed of 2.10% (*m*/*v*) of cholesterol (Sigma^®^, St. Louis, MO, USA), 1.70% (*m*/*v*) of lipoid E80^®^ (Lipoid^®^, Ludwigshafen, Germany), and 96.2% (*m*/*v*) of 1-octanol (Synth^®^, São Paulo, SP, Brazil) [49,50]. The choice of this composition is justified by its ability to simulate the barrier function of the lipid matrix of the stratum corneum [51]. Cholesterol increases the rigidity of the lipid phase, the phospholipids present in Lipoid E80 promote amphiphilic organization, and the 1-octanol used as a solvent ensures homogeneous impregnation and presents a lipophilic/hydrophilic balance with a solubility parameter similar to that of biological membranes [50,52,53].

The receiving compartment was filled with PBS buffer solution and 2.5% Tween 80 (pH 7.4), maintained at 32 ± 1 °C under constant agitation at 300 rpm (AMP-10 Multi-Position Shaker, Gehaka, São Paulo, Brazil). Approximately 0.3 g of the formulations (F1, F2, F7, and F8) was applied to the donor compartment. Samples (1 mL) were collected at 0, 1, 2, 3, 4, 6, 8, and 24 h, with the same volume replaced in the receiver compartment to maintain sink conditions.

The release of FA and CF was quantified using a previously validated chromatographic method on an Ultra Performance Liquid Chromatography (UPLC) system (Shimadzu^®^, Kyoto, Japan), operated with LC Solution software (v 1.25). The mobile phase consisted of acetonitrile and acidified water [54].

The cumulative amount of FA released was expressed in μg/cm^2^ and plotted as a function of time. The percentage of release was calculated using the equation [55,56]:

(3) $$Release\;(\%)=\frac{Rt}{Q\times 100}$$

where *Rt* represents the cumulative amount released at time *t*, and *Q* represents the initial amount of the active ingredient in the formulation.

The in vitro skin release data were fit to zero-order (µg/cm^2^ versus time), first-order (log µg/cm^2^ versus time), and Higuchi (µg/cm^2^ versus square root of time) kinetic models. The model that best described the release profile was determined by linear regression based on the R^2^ value closest to 1.

The flow of FA through the membranes in the steady state (J) was determined from the slope of the line obtained in the linear region of the curve after diffusion equilibrium. The lag time, defined as the time required for the drug to cross the membrane and reach steady state, was estimated as the time at which the extrapolated steady-state line intersected the time axis. The apparent permeability coefficient (P) was calculated according to the equation:

(4) $$P=\frac{J}{C0}$$

where (C0) represents the initial concentration of the drug in the formulation.

### 2.7. Statistical Analyses

The results were expressed as mean and standard deviation. Statistical analysis of the data was performed using GraphPad Prism 8.0.2 software (GraphPad Software Inc., La Jolla, CA, USA). Statistical significance was determined by analysis of variance (ANOVA), followed by Tukey’s post hoc test (*p* < 0.05).

## 3. Results and Discussion

### 3.1. Physical and Chemical Characterization

#### 3.1.1. FTIR

**Figure 1:**
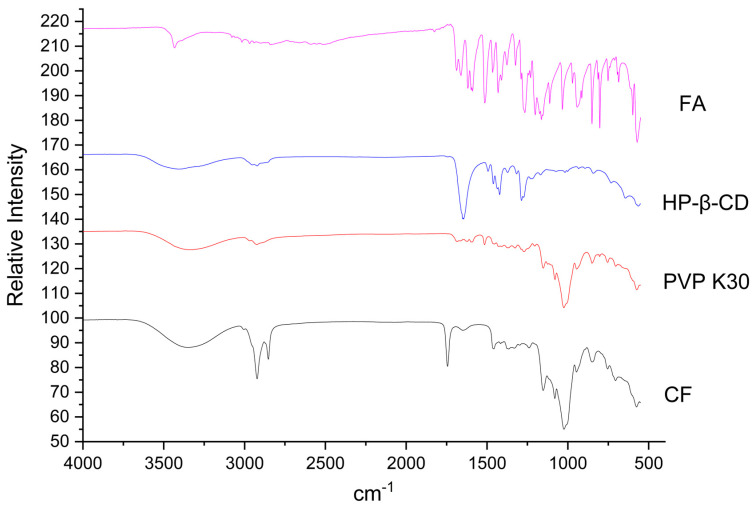
Infrared spectra of Ferulic Acid (FA), Hydroxypropyl β-cyclodextrin (HPβ-CD), polyvinylpyrrolidone K30 (PVP K30), and Cicloferulic^®^ (CF).

FTIR spectroscopy confirmed the formation of a multicomponent system comprising FA, HP-β-CD, and PVP K30 (Figure 1).

In the FA spectrum, a broad band at 3430 cm^−1^ attributed to O–H stretching is observed, followed by peaks at 2838 cm^−1^ and between 1687 and 1660 cm^−1^, corresponding to C–H stretching and C=O conjugated to the aromatic ring, respectively. The bands at 1619, 1591, 1511, 1431, and 1202 cm^−1^ are typical of aromatic ring vibration, while the band at 1266 cm^−1^ corresponds to asymmetric C–O–C stretching. The signals at 850 and 801 cm^−1^ indicate hydrogens adjacent to the phenolic ring [57,58,59].

The HP-β-CD spectrum shows characteristic bands around 2930 cm^−1^ (O–H and C–H stretching), 1650 cm^−1^ (ring stretching), and 1370 cm^−1^ (O–H and CH_3_ bending), as well as C–C and C–O vibrations at 1028, 1150, and 1180 cm^−1^, including the antisymmetric C–O–C glycosidic bridge [59,60,61,62]. PVP K30 is characterized by C–H stretching at 2931 cm^−1^, C=O stretching at 1645 cm^−1^, and a broad band at 3415 cm^−1^ associated with the presence of water [23,63].

The CF spectrum is similar to the HP-β-CD profile because of its predominant composition. FA bands appear shifted and with lower intensity, such as the shift from 1619, 1511, 1431 to 1632, 1516, 1428 cm^−1^; the 1687–1660 cm^−1^ region has been converted into a single band between 1638 and 1655 cm^−1^, and typical FA signals at 1591 and 1202 cm^−1^ have disappeared.

The asymmetric C–O–C stretch was shifted from 1266 to 1272 cm^−1^. The broad bands of HP-β-CD (3324–3355 cm^−1^) merged with the O–H of FA. The PVP K30 signals were masked by cyclodextrin absorption due to the low polymer concentration in the system. These effects of band shift, disappearance, and broadening indicate interaction between the components and the formation of an inclusion system, with the aromatic ring of FA likely inserted into the hydrophobic cavity of CD. At the same time, polar groups remain exposed on the surface [57,64,65].

#### 3.1.2. Thermal Analysis

##### DSC

**Figure 2:**
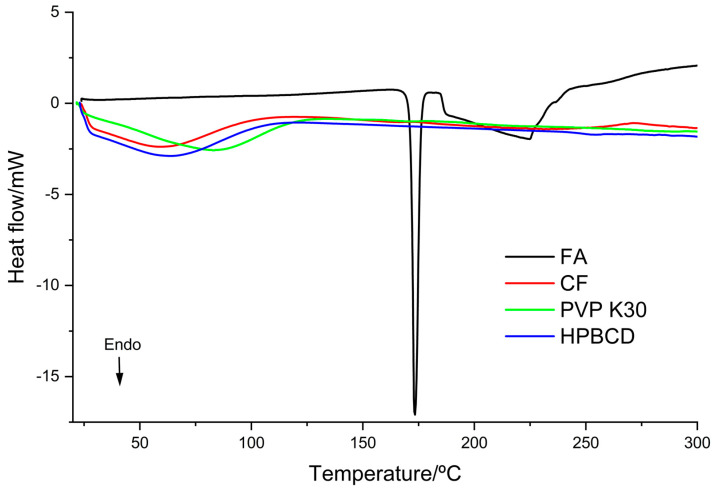
DSC curves of isolated components and a multicomponent system at a heating rate of 10 °C/min: Ferulic Acid (FA), Hydroxypropyl β-cyclodextrin HPβCD, Cicloferulic^®^ (CF), and Polyvinylpyrrolidone K30 (PVP K30).

Differential scanning calorimetry (DSC) analysis revealed significant differences between the isolated components and CF (Figure 2).

FA showed a clear endothermic peak at approximately 168 °C, corresponding to melting, followed by an exothermic event associated with decomposition, characteristic of its crystalline structure and lower thermal stability. The PVP K30 polymer exhibited an endothermic dehydration peak near 100 °C and a broad exothermic event above 450 °C, indicating high thermal stability.

For HPβCD, we observed an endothermic peak around 100 °C, attributed to dehydration, and subsequent exothermic events of crystallization and decomposition between 263 °C and 615 °C, with high enthalpies, behavior consistent with its molecular stability [60,66]. The extent of decomposition up to about 615 °C can be explained by additional stages of thermal degradation, related to the formation of a structurally stable carbonaceous residue (char) [67,68].

The CF showed overlapping HPβCD peaks and the absence of the characteristic FA melting peak, indicating effective complexation of the active ingredient. The thermal profile of the CF showed smoother transitions, peak shifts to lower temperatures, and reduced enthalpies, indicating loss of ferulic acid crystallinity and strong molecular interaction between FA, HPβCD, and PVP K30. These findings reinforce the formation of a stable multicomponent system, with FA encapsulated in the cyclodextrin cavity and interacting with the polymer, resulting in reduced crystallinity and modification of the active ingredient’s thermal behavior, as reported in previous studies [59,69].

##### TG/DTG

**Figure 3:**
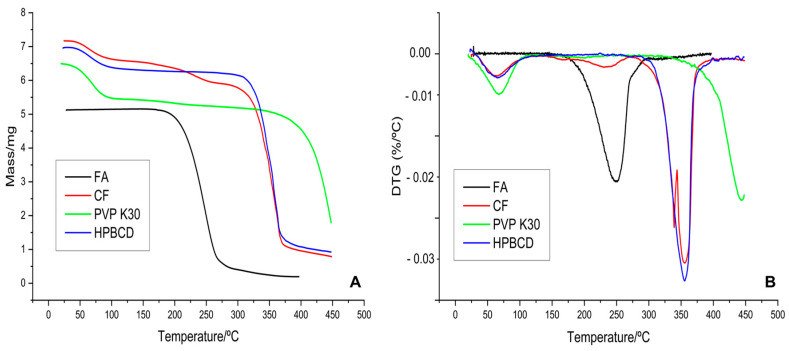
(**A**) TG and (**B**) DTG curves of Ferulic Acid (FA), Hydroxypropyl β-cyclodextrin HPβCD, Cicloferulic^®^ (CF), and Polyvinylpyrrolidone K30 (PVP K30), at a rate of 10 °C/min.

The thermogravimetric analysis (TG/DTG) shown in Figure 3 reveals that FA undergoes three distinct decomposition stages, with the process beginning at approximately 153 °C.

HP-β-CD exhibited initial mass loss between 31 and 106 °C, attributed to the elimination of adsorbed water due to its hygroscopicity, and a main degradation event between 289 and 377 °C. For PVP K30, the thermal profile showed similar initial dehydration; however, the polymer demonstrated greater thermal stability, with marked degradation near 367 °C, in agreement with previous studies [70]. CF showed a shift in the main degradation event of FA to lower temperatures (124–269 °C) and a predominant mass loss around 269 °C, suggesting changes in molecular interactions and a relevant modification in the thermal behavior of the active ingredient. These results indicate the formation of a multicomponent system, with significant changes and improvement in the thermal stability of the isolated components [71,72].

#### 3.1.3. DRX

**Figure 4:**
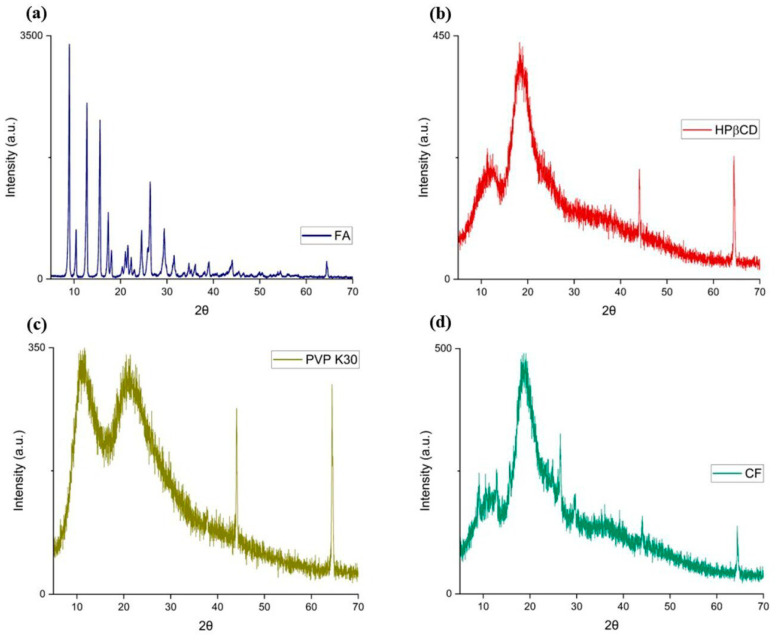
Diffractograms of (**a**) Ferulic Acid (FA), (**b**) Hydroxypropyl β-cyclodextrin HPβCD, Polyvinylpyrrolidone K30 (PVP K30) (**c**), and (**d**) Cicloferulic^®^ (CF).

The X-ray diffractogram of FA showed intense, well-defined crystalline reflections, indicating its crystalline nature (Figure 4a).

The prominent crystalline reflections were observed at 8.85°, 14.57°, 17.75°, 26.98°, and 30.61°, consistent with the profile reported in previous studies and with the P21/n space group of the Cambridge Crystallographic Data Center (CCDC) (GASVOL) [59,73]. PVP K30 presented a diffractometric pattern characteristic of a predominantly amorphous material, with a diffuse halo and absence of marked crystalline reflections. However, some discrete reflections may indicate traces of residual crystallinity (Figure 4c). The HPβCD spectrum showed semicrystalline characteristics, with moderate intensity crystalline reflections superimposed on amorphous halo regions, suggesting a partially amorphous structure (Figure 4b). For CF, we observed the absence of the characteristic FA peaks, resulting in a predominantly amorphous pattern and a diffuse halo similar to that of cyclodextrin (Figure 4d). This result indicates a new molecular organization, compatible with interaction and inclusion of FA in the HPβCD cavity [74]. The reduction in crystallinity observed in the system represents a significant advance, as it is associated with improved solubility and a potential increase in the bioavailability of encapsulated ferulic acid [59,75,76].

#### 3.1.4. Reduction in the Radical 2,2-Diphenyl-1-Picrylhydrazil (DPPH)

**Figure 5:**
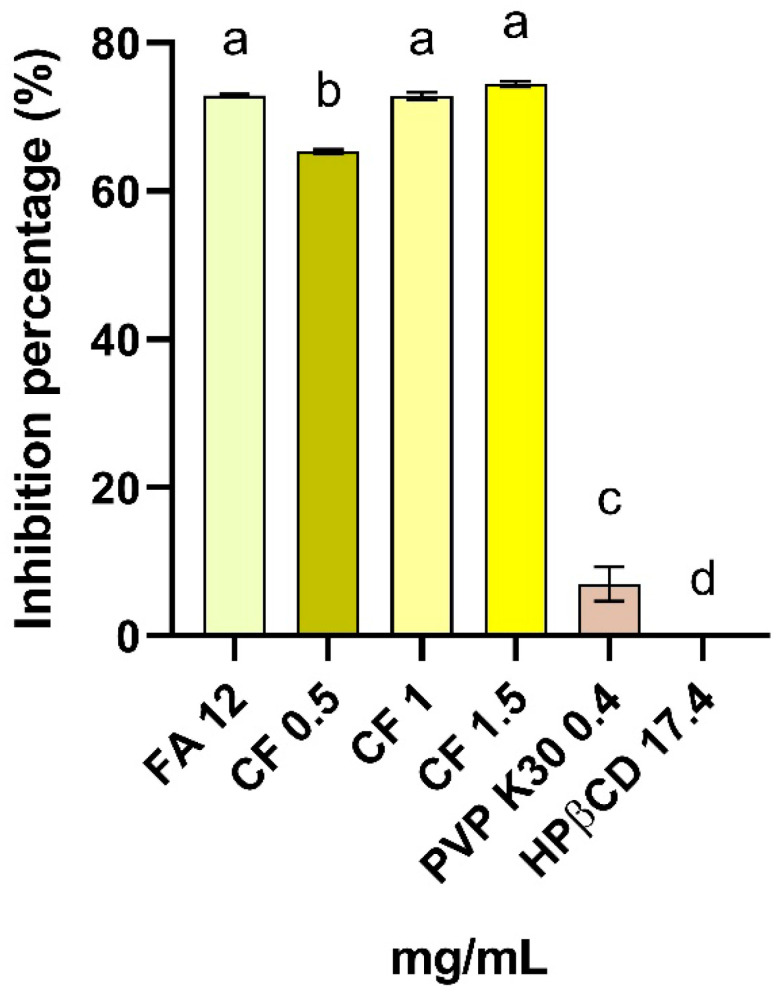
Antioxidant activity of Ferulic Acid (FA), Cicloferulic^®^ (CF), Hydroxypropyl β-cyclodextrin HPβCD and Polyvinylpyrrolidone K30 (PVP K30) by DPPH●. Different letters indicate a statistically significant difference (*p* < 0.05).

The evaluation of the antioxidant activity of FA revealed a DPPH free radical inhibition percentage of 72.82% at a concentration of 12 mg/mL (Figure 5).

This result demonstrates the high efficiency of FA as an antioxidant agent, highlighting its ability to neutralize free radicals. CF showed DPPH radical scavenging capacity of 65.35%, 72.52%, and 74.61% at concentrations of 0.5 mg/mL, 1 mg/mL, and 1.5 mg/mL, respectively. Considering that the multicomponent system contained 0.1875 mg/mL of FA at the highest concentration evaluated (1.5 mg/mL), CF showed a similar reduction percentage (74.61%) to that of FA (12 mg/mL: 72.82%), despite the significantly lower FA concentration in the system. The other components of CF, HP-β-CD and PVP K30, did not show significant antioxidant activity.

Thus, we can infer that the arrangement and molecular structure of CF enhanced its antioxidant effect by interacting with reactive oxygen species, thereby conferring superior antioxidant properties [77,78].

The primary mechanism responsible for the antioxidant activity of FA is the donation of a proton by the phenolic group, forming a phenoxy radical stabilized by resonance within the aromatic ring and the conjugated side chain –CH=CH–COOH [79,80]. In this context, the complexation of FA enhances its activity by promoting its solubilization and the formation of intermolecular hydrogen bonds between the host molecule and the DC, thereby increasing the availability and stability of the active antioxidant form [81,82].

We calculated the IC_50_ to determine the appropriate concentration for incorporating CF into cosmetic formulations. The concentration of the active ingredient required to inhibit 50% of the DPPH radical was 311.5476 µg/mL. Based on this result, we selected a CF concentration of 500 µg/mL (0.5% *w*/*w*) for incorporation into cosmetic formulations.

### 3.2. In Vitro Cytotoxicity Assessment

**Figure 6:**
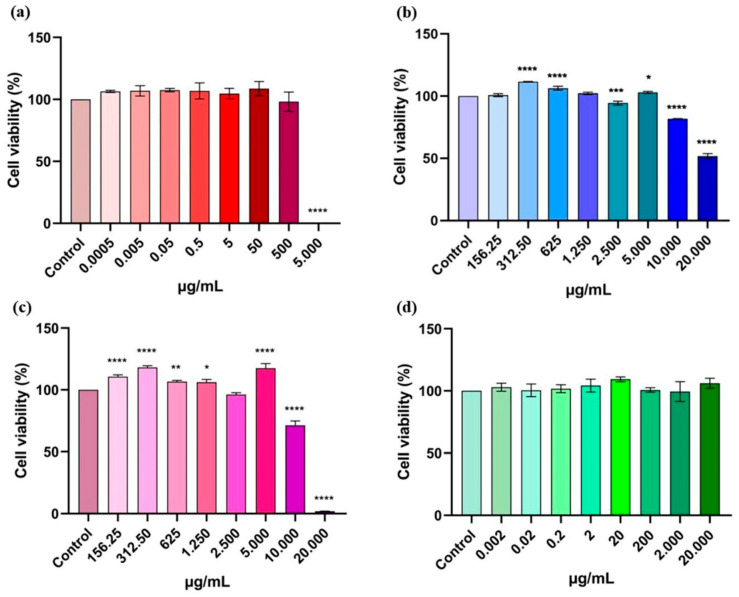
Cell viability of human HaCaT keratinocytes treated with (**a**) Ferulic Acid (FA)-(**** *p* < 0.0001), (**b**) Hydroxypropyl β-cyclodextrin (HPβCD)-(**** *p* < 0.0001); *** *p* = 0.0001; * *p* = 0.0316), (**c**) Cicloferulic^®^ (CF)-(**** *p* < 0.0001; ** *p* = 0.0072; * *p* = 0.0123), and (**d**) Polyvinylpyrrolidone K30 (PVP K30). Asterisks indicate a statistically significant difference between the tested concentrations and the control.

In vitro cytotoxicity assays using the HaCaT keratinocyte cell line are an essential tool for assessing the safety and biological compatibility of various topical active ingredients [83,84]. At the FA concentrations we tested, ranging from 0.0005 µg/mL to 500 µg/mL, there was no significant change in cell viability compared to the control, remaining at approximately 100% (Figure 6a).

For values above the IC_50_ (1.402 ± 0.716 μg/mL), such as the highest FA concentration tested (5000 µg/mL), we observed a reduction in cell viability of up to 100%, indicating a cytotoxic effect under these conditions. Possibly, the change in medium pH caused by the high FA concentration compromised cell integrity, affecting processes such as plasma membrane function and homeostatic balance, which may have induced stress and cell death [85,86]. The buffered cell medium (pH 7.4) maintained a relatively stable pH at lower FA concentrations; however, at higher concentrations, the buffering capacity was insufficient to neutralize the acidification induced by the compound [87,88].

For HPβCD, we observed no cytotoxicity at concentrations up to 10.000 µg/mL, with cell viability remaining close to 100% compared to the control, indicating a safe profile within this concentration range. Only at the highest concentration (20.000 µg/mL) did we observe a significant reduction in cell viability (≈51.7%; *p* < 0.05), suggesting a cytotoxic effect at concentrations higher than those typically used in cosmetic formulations (Figure 6b). Studies have shown that HPβCD exerts a cytotoxic influence on cell proliferation at concentrations above 0.5% and 1% (*w*/*v*) [89]. The cytotoxicity of cyclodextrins is widely attributed to their ability to complex lipophilic substances in cell membranes, thereby compromising their integrity [90,91]. The IC_50_ was 11.68 ± 0.65 µg/mL, and concentrations below this value are safe and compatible with topical applications.

For PVP K30 (Figure 6d), we observed no significant changes in cell viability at any concentration tested relative to the control, demonstrating the polymer’s safety within the concentration range studied.

For CF (Figure 6c), cell viability was maintained up to a concentration of 5.000 µg/mL, demonstrating a safety profile within this range. At a concentration of 10.000 µg/mL, we observed a significant reduction in cell viability to 71.25%. In contrast, at 20.000 µg/mL, viability was reduced entirely, indicating a cytotoxic effect only at very high exposure levels. The IC_50_ we calculated was 10.075 ± 0.377 µg/mL, suggesting that Cicloferulic^®^ is safe at the concentration used in the cosmetic formulations we developed in this study.

The higher safety profile of CF compared to FA may be related to the fact that CF contains only 12% FA in its composition. The cytotoxicity we observed at higher concentrations of CF may be attributed to the cyclodextrin used as an excipient, which constitutes the majority of the system. These results highlight the importance of individually evaluating the components and the multicomponent system for safe cosmetic applications.

### 3.3. Antioxidant Activity in Cells

**Figure 7:**
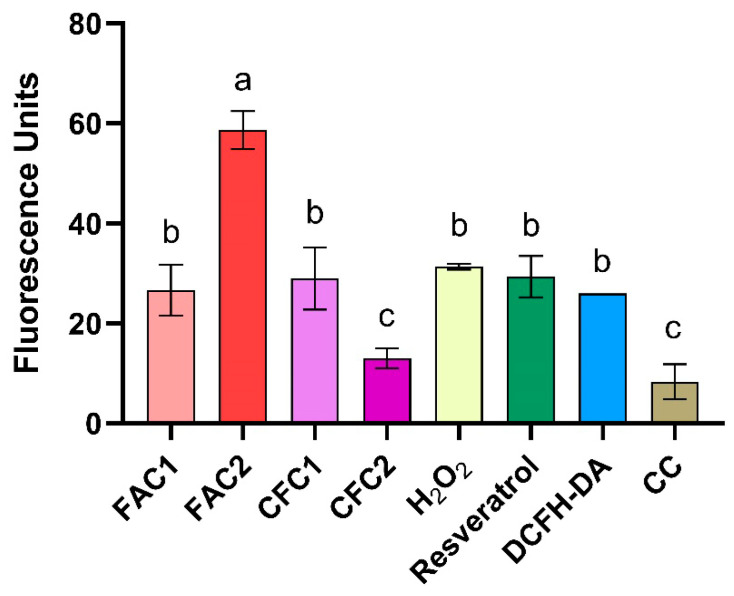
Antioxidant activity of FA (Ferulic Acid) and CF (Cicloferulic^®^) in human keratinocyte cells, HaCaT line. FA (0.6 mg/mL), FA (60 mg/mL), CF (0.6 mg/mL), CF (60 mg/mL); H_2_O_2_: Oxidant; Resveratrol: Positive control; DCFH-DA (2′,7′-dichlorofluorescein diacetate): Dye; CC: Cell control. Different letters indicate statistically significant differences (*p* < 0.05).

In the antioxidant activity assay in HaCaT cells, we evaluated FA and CF at concentrations of 0.6 mg/mL (IC_10_) and 60 mg/mL (Figure 7).

For FA (0.6 mg/mL), there was no significant difference (*p* > 0.05) in the fluorescence index between resveratrol and FA (0.6 mg/mL). However, in FA (60 mg/mL), we observed a significant reduction (*p* < 0.05) in antioxidant activity compared to the control, attributed to the pro-oxidant effect of antioxidants at high concentrations [92]. Phenolic compounds, such as FA, can catalyze reactions that generate harmful radicals, such as Fenton reactions, by reducing transition metal ions present in the cellular environment [93,94]. An excess of reducing equivalents can lead to reductive stress, which can induce increased ROS production and, consequently, cellular stress [95]. In addition, the mean inhibitory concentration (IC_50_) for FA cytotoxicity was 1.402 ± 0.716 µg/mL, indicating that FA is cytotoxic at 60 mg/mL, as observed in this study. This effect also explains the reduction in antioxidant activity, as cellular toxicity compromises antioxidant mechanisms, leading to the generation of reactive species and reducing the effectiveness of the active ingredient [96].

When comparing FA (0.6 mg/mL) and CF (0.6 mg/mL), there was no significant difference (*p* > 0.05), indicating similar antioxidant activity between samples at the same concentration, despite CF containing 0.072 mg/mL of FA (equivalent to 12% FA in the multicomponent system), suggesting that complexation enhances ROS neutralization, allowing a smaller amount of FA to exhibit antioxidant efficacy equivalent to or greater than that of the isolated compound [97]. CF (0.6 mg/mL) also showed activity similar to that of the positive control (*p* > 0.05).

In CF (60 mg/mL), we observed antioxidant activity superior to FA and the positive control. Even at high concentrations, CF did not show pro-oxidant behavior like FA (60 mg/mL), possibly because, although the CF concentration is high, the amount of FA available in the cellular medium (7.2 mg/mL, equivalent to 12% of FA in the system) remains within the safe range in which FA acts predominantly as an antioxidant, without inducing cellular stress or pro-oxidant effects. In addition, complexation provides structural protection to FA, reducing its direct exposure to the cellular environment and improving its stability and antioxidant performance [77]. The formation of intermolecular hydrogen bonds between FA and CD may have contributed to the improvement in antioxidant efficacy, suggesting that this molecular organization favors more efficient interactions with free radicals, thereby amplifying the antioxidant response [78,79,81,82].

Thus, the data indicate that the use of CF not only optimizes the antioxidant activity of FA but also enables the use of significantly lower amounts of this active ingredient to achieve the same or better effect, thereby reducing the formulation’s cost without compromising its effectiveness. This aspect is advantageous in cosmetic applications, where the efficacy of the active ingredient and the cost–benefit ratio of the final product are determining factors in developing innovative and safe formulations.

### 3.4. In Vitro Photoprotective Efficacy and Photostability Test

**Figure 8:**
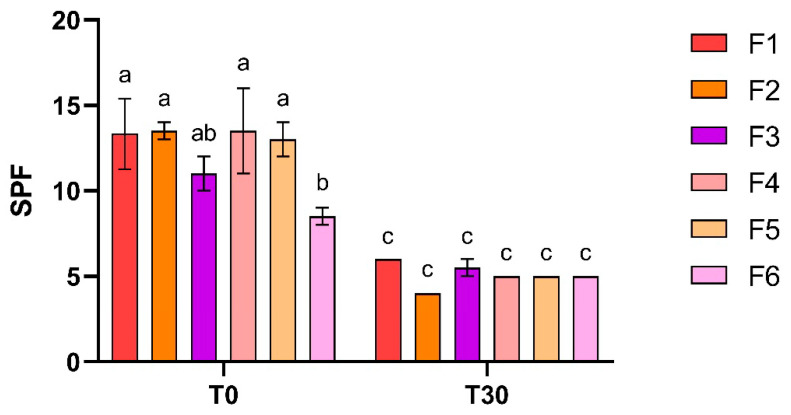
SPF of cosmetic formulations at times T0 and T30. F1: formulation with FA and sunscreens (5% butyl methoxydibenzoylmethane and 10% ethylhexyl methoxycinnamate); F2: formulation with CF and sunscreens (5% butyl methoxydibenzoylmethane and 10% ethylhexyl methoxycinnamate); F3: formulation with sunscreens (5% butyl methoxydibenzoylmethane and 10% ethylhexyl methoxycinnamate); F4: formulation with FA and sunscreens (2.5% butyl methoxydibenzoylmethane and 5% ethylhexyl methoxycinnamate); F5: formulation with CF and sunscreens (2.5% butyl methoxydibenzoylmethane and 5% ethylhexyl methoxycinnamate); F6: formulation with sunscreens (2.5% butyl methoxydibenzoylmethane and 5% ethylhexyl methoxycinnamate). Different letters indicate a statistically significant difference (*p* < 0.05). Where error bars are not visible, the standard deviation is zero.

The results of the in vitro photoprotective efficacy assessment of the formulations (F1, F2, F3, F4, F5, and F6) are shown in Figure 8, expressing the sun protection factor (SPF) values before exposure to radiation (T0) and after 30 min of artificial UV irradiation (T30).

Formulations F7 and F8 did not contain sunscreens and presented SPF values close to 1. In Figure 8, we can see that formulations F1, F2, F4, and F5 present SPF values between 13.0 and 13.5 (*p* > 0.05). The results demonstrated increased photoprotective efficacy with the addition of antioxidant active ingredients. Formulations F4 and F5 had SPF values equivalent to formulations F1 and F2, which contained twice the concentration of organic sunscreens. These data demonstrate that the addition of the antioxidant active ingredients FA and CF acted synergistically with the sunscreens, contributing to greater photoprotective efficacy of the formulations studied, reinforcing the multifunctional potential of antioxidants in line with the results obtained in this study [9,97,98,99,100]. These results are promising for the development of more sustainable photoprotectors, as they enable a reduction in the amount of chemical filters in the formulation, also contributing to a lower risk of skin irritation, greater sensory compatibility, and cost reduction, without compromising photoprotective efficacy [101].

In formulations F3 and F6, which did not contain FA and CF, the SPF value was significantly lower when compared to formulations F1, F2, F4, and F5, reinforcing that the presence of antioxidant active ingredients plays a fundamental role in increasing SPF, enabling the reduction in filters and confirming their functional effect.

The SPF and photostability results (Table 2 and Figure 8) indicate that after 30 min of irradiation (T30), all formulations showed a significant reduction in SPF values (4–6) compared to T0. The formulations (F1, F2, F4, F5) showed reductions of up to 70% in SPF, with no significant difference (*p* > 0.05), indicating that, although they contribute to the initial increase in photoprotection, the antioxidants evaluated were not sufficient to stabilize systems containing intrinsically unstable sunscreens [102,103,104,105].

Butyl methoxychinamate is an efficient UVA filter, but it is susceptible to photolysis, isomerization, or decomposition when exposed to UV radiation [106,107]. In addition, although ethylhexyl methoxychinamate is more stable, studies show that when combined with butyl methoxychinamate, it absorbs UVA energy and transfers part of this energy to ethylhexyl methoxycinnamate, promoting its photolysis and generating reactive species, which accelerates the mutual degradation of the filters, rapidly reducing the protection capacity and photostability of the formulation in the absence of effective stabilizers [102,108]. There were limitations in the formulations evaluated, evidenced by reductions in SPF values after UV irradiation, without the tested active ingredients contributing effectively to the system’s photostability. Future formulations incorporating CF may require the addition of photostabilizers, such as triplet-state quenchers that act via triplet-triplet energy transfer, as well as combination with other antioxidants, such as quercetin. Such approaches may help mitigate photolysis and improve the photostability of photoprotective formulations [109,110].

Even so, this study pioneers the use of the multicomponent system, demonstrating that CF not only enhances antioxidant activity but also contributes to photoprotection. These results highlight the innovative nature and technological potential of CF for cosmetic applications.

### 3.5. In Vitro Study of Skin Release of Cosmetic Formulations

**Figure 9:**
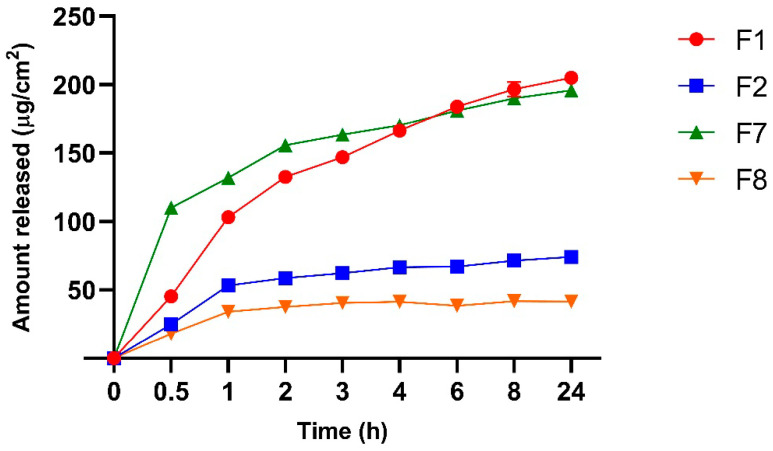
In vitro release profiles of FA (Ferulic acid) from formulations F1, F2, F7, and F8. F1: formulation with FA and sunscreens (5% butyl methoxydibenzoylmethane and 10% ethylhexyl methoxycinnamate); F2: formulation with CF (Cicloferulic^®^) and sunscreens (5% butyl methoxydibenzoylmethane and 10% ethylhexyl methoxycinnamate); F7: formulation with FA and no sunscreens; F8: formulation with CF and no sunscreens.

To evaluate the influence of sunscreens on the release of FA and CF, we tested formulations containing the highest concentrations of sunscreens (5% butyl methoxydibenzoylmethane and 10% ethylhexyl methoxycinnamate). The release profiles (*p* < 0.05) are shown in Figure 9.

In formulations containing FA, F1 showed slower initial release than F7, with a significant difference (*p* < 0.05) at all time points except 4 h. This behavior may be associated with the nonpolar nature of F1, due to the presence of sunscreens, which increase the affinity of FA for the vehicle relative to F7, a less nonpolar medium [111,112,113]. After 6 h, however, we observed greater release of FA in F1, possibly due to the high lipophilicity of sunscreens, which initially promote greater solubilization and a higher free fraction of the active ingredient, favoring the diffusion of FA. We also consider that the affinity of these compounds for the membrane used, impregnated with lipophilic constituents, may, over time, lead to its saturation or structural alteration, reducing the subsequent diffusion of FA. Thus, the dynamic interaction between sunscreens and the membrane may explain the observed biphasic kinetics, highlighting the influence of the formulation composition and diffusion barrier on the release profile [114].

In formulations containing CF, F2 showed greater FA release than F8, with significant differences (*p* < 0.05) at all time points evaluated. The molecular interaction between sunscreens and the cyclodextrin cavity may have been a determining factor for the observed behavior. The affinity between molecules and the cyclodextrin cavity depends on factors such as binding constant (Ka), lipophilicity (logP), and molecular interactions [115]. More lipophilic molecules, such as sunscreens (logP = 4.5 to 6.0), have a greater affinity for the nonpolar cavity of CD than FA (logP = 1.3 to 1.5), resulting in competition for the cavity and displacement of FA to the medium, thus increasing its availability, as observed in F2 [69,113,116]. In contrast, F8 showed a lower release profile, probably due to the greater affinity of FA for the cyclodextrin cavity, at the expense of the formulation’s aqueous vehicle [18,111,117].

In previous work, Sánchez-Campillo et al. (2009) observed that rosmarinic acid (RA) did not permeate the epidermis in detectable amounts, suggesting that its antioxidant action occurs mainly on the skin surface, a desirable characteristic in cosmetic formulations, since it avoids systemic absorption and potential adverse effects [118]. Similarly, Psotova et al. (2006) investigated the photoprotective properties of RA in a human keratinocyte cell line (HaCaT), demonstrating that the compound reduced UVA-induced damage [119]. This protection is related to the preservation of cell viability, attributed to RA’s antioxidant and anti-inflammatory properties, which neutralize reactive oxygen species (ROS) generated during exposure to ultraviolet radiation [120]. RA and FA have similar chemical structures and logP values, with phenolic and carboxylic groups responsible for hydrogen donation and free radical neutralization [120,121]. Thus, the retention of FA in the Cicloferulic^®^ multicomponent system observed in F8 confirms the effectiveness of CF in modulating and modifying the bioavailability of FA, favoring a safer topical action and contributing to the neutralization of ROS generated by UV radiation on the skin surface. These findings also highlight the importance of formulation composition on the release profile, showing that sunscreens not only affect release but also influence the behavior of the multicomponent system, altering the interaction between FA and cyclodextrin [111]. Formulations F2 and F8 release less FA than F1 and F7, reinforcing the influence of the binding strength of FA to the cyclodextrin cavity, thereby reducing its availability in the medium [111,117,122].

We analyzed the data in zero-order (µg/cm^2^ versus time), first-order (log µg/cm^2^ versus time), and Higuchi (µg/cm^2^ versus square root of time) models. We selected the most appropriate kinetic model based on the R^2^ value closest to 1 obtained by linear regression, as shown in Table 3.

The kinetic profile of FA release in formulations F1 and F7 best fit the Higuchi kinetic model. Formulations CF, F2, and F8 exhibited release profiles influenced by the diffusion of the active ingredient and by molecular interactions within the system, which altered the apparent solubility of the active ingredient and possibly modified the formulation’s physicochemical properties. These interactions favor a more gradual, continuous release, consistent with the modified Higuchi model [123]. The complexation of FA modulates the release rate, increasing the retention capacity of the active ingredient and delaying its diffusion, implying a physicochemical rearrangement of the matrix that results in a more complex release mechanism [124]. Thus, the release profile we observed indicates a balance between the diffusion of FA and the structural changes induced by complexation and excipients, which explains the behavior of complexed formulations, in contrast to the faster release profile observed in formulations with FA [125,126,127].

We determined the steady-state flow rate of FA (J), lag time (T_L_), and permeability coefficient (P) for each experimental condition using the kinetic model, as shown in Table 4.

In F1 and F7, we observed that F7 presented greater release and flow of the active ingredient in less time, possibly due to the hydrophobic nature of FA and its low affinity for the vehicle [128,129,130]. In general, both exhibited high flow rates and permeability coefficients, with virtually zero lag time, indicating rapid availability and diffusion of FA through the membrane. On the other hand, F2 and F8 showed reduced J and P, with increased lag time. FA complexation tends to increase apparent solubility in the vehicle, but reduces the free fraction available for diffusion [122].

In general, formulations with FA released the active ingredient more efficiently than those containing CF, due to faster diffusion into the receiving medium, as demonstrated by the kinetic parameters. However, the decrease in release flux observed in formulations with CF may be a strategic feature because it provides more controlled bioavailability of FA, ensuring that photoprotective and antioxidant formulations exert a safe action on the skin surface.

Although the lipid-impregnated cellulose acetate membrane provides a controlled system for comparative release assessment, it represents a simplified artificial model. It does not reproduce the structural and biochemical complexity of human skin. Therefore, additional studies using ex vivo or in vivo skin models are necessary to confirm the translatability and biological relevance of these findings.

## 4. Conclusions

Cicloferulic^®^ demonstrated superior antioxidant activity to ferulic acid and maintained a high safety profile in HaCaT cells. In photoprotective formulations, CF increased SPF and enabled a reduction in the amount of UV filters without loss of efficacy, while also modifying in vitro skin release. However, after irradiation, formulations with CF showed a significant reduction in SPF, indicating the need for further studies to improve the stability of intrinsically unstable sunscreens. These findings, unprecedented in the literature, position CF beyond conventional systems limited to solubility enhancement, supporting its role as a multifunctional system that enhances the antioxidant and photoprotective actions of FA, contributing to the development of safer, more effective cosmetics in line with the search for more sustainable solutions.

## Figures and Tables

**Table 1:** Composition of cosmetic formulations evaluated for Sun Protection Factor (SPF).

Ingredients	Concentration (% *w*/*w*)							
	F1	F2	F3	F4	F5	F6	F7	F8
Polyacrylamide (and) C13-14 Isoparaffin (and) Laureth-7	3.5	3.5	3.5	3.5	3.5	3.5	3.5	3.5
Dissodium EDTA	0.1	0.1	0.1	0.1	0.1	0.1	0.1	0.1
Phenoxyethanol (and) methylparaben (and) ethylparaben (and) butylparaben (and) propylparaben (and) isobutylparaben	0.5	0.5	0.5	0.5	0.5	0.5	0.5	0.5
Propylene Glycol	3	3	3	3	3	3	3	3
Butyl methoxydibenzoylmethane	5	5	5	2.5	2.5	2.5		
Ethylhexyl methoxycinnamate	10	10	10	5	5	5		
Cicloferulic^®^		0.5			0.5			0.5
Ferulic acid	0.06			0.06			0.06	
Purified water (qsp)	100	100	100	100	100	100	100	100

F1: formulation with FA (Ferulic acid) and sunscreens (5% butyl methoxydibenzoylmethane and 10% ethylhexyl methoxycinnamate); F2: formulation with CF (Cicloferulic^®^) and sunscreens (5% butyl methoxydibenzoylmethane and 10% ethylhexyl methoxycinnamate); F3: formulation with sun filters (5% butyl methoxydibenzoylmethane and 10% ethylhexyl methoxycinnamate); F4: formulation with FA and sun filters (2.5% butyl methoxydibenzoylmethane and 5% ethylhexyl methoxycinnamate); F5: formulation with CF and sunscreens (butyl methoxydibenzoylmethane 2.5% and ethylhexyl methoxycinnamate 5%); F6: formulation with sunscreens (butyl methoxydibenzoylmethane 2.5% and ethylhexyl methoxycinnamate 5%); F7: formulation with FA and no sunscreens; F8: formulation with CF and no sunscreens.

**Table 2:** Sun protection factor (SPF in vitro), critical wavelength (nm), and percentage of SPF decay after irradiation for formulations F1 to F6.

Formulations	Pre-Irradiation		Post-Irradiation (15 min)		SPF Decay (%)	Post-Irradiation (30 min)		SPF Decay (%)
	SPF	Critical Wavelength (nm)	SPF	Critical Wavelength (nm)		SPF	Critical Wavelength (nm)	
F1	13.33 ± 1.70	381.67 ± 0.58	8.0 ± 0	381.67 ± 0.58	42.86	6.0 ± 0	381.67 ± 0.58	54.20
F2	13.50 ± 1.7	381.33 ± 0.58	6.0 ± 0	382 ± 0.00	55.51	4.0 ± 0	382 ± 0.00	70.34
F3	11.0 ± 0.82	381.33 ± 0.58	7.5 ± 0.41	382.33 ± 0.58	31.57	5.5 ± 0.41	382 ± 0.00	49.85
F4	13.50± 2.04	380 ± 0	6.0 ± 0	379.67 ± 0.58	54.50	5.0 ± 0	378.67 ± 0.58	62.09
F5	13.0 ± 0.82	379 ± 0	6.0 ± 0	379.67 ± 0.58	53.66	5.0 ± 0	378.33 ± 0.58	61.39
F6	8.5 ± 0.41	380 ± 0	6.0 ± 0	379 ± 0.00	29.25	5.0 ± 0.47	378 ± 0	41.04

F1: formulation with FA (Ferulic acid) and sunscreens (5% butyl methoxydibenzoylmethane and 10% ethylhexyl methoxycinnamate); F2: formulation with CF (Cicloferulic^®^) and sunscreens (5% butyl methoxydibenzoylmethane and 10% ethylhexyl methoxycinnamate); F3: formulation with sun filters (5% butyl methoxydibenzoylmethane and 10% ethylhexyl methoxycinnamate); F4: formulation with FA and sun filters (2.5% butyl methoxydibenzoylmethane and 5% ethylhexyl methoxycinnamate); F5: formulation with CF and sunscreens (butyl methoxydibenzoylmethane 2.5% and ethylhexyl methoxycinnamate 5%); F6: formulation with sunscreens (butyl methoxydibenzoylmethane 2.5% and ethylhexyl methoxycinnamate 5%).

**Table 3:** Coefficients of determination (R^2^) of kinetic models used to fit the release profile of FA.

Formulation	Zero Order (R^2^)	First Order (R^2^)	Higuchi (R^2^)
F1	0.8585	0.5441	0.9603
F2	0.9627	0.6775	0.9925
F7	0.8701	0.7777	0.9821
F8	0.9353	0.6703	0.9994

F1, F2, F7, and F8. F1: formulation with FA (Ferulic acid) and sunscreens (5% butyl methoxydibenzoylmethane and 10% ethylhexyl methoxycinnamate); F2: formulation with CF (Cicloferulic^®^) and sunscreens (5% butyl methoxydibenzoylmethane and 10% ethylhexyl methoxycinnamate); F7: formulation with FA and no sunscreens; F8: formulation with CF and no sunscreens.

**Table 4:** Kinetic parameters obtained from in vitro FA release tests.

Parameters	F1	F2	F7	F8
J (μg cm^−2^ h^−1^)	90.49	15.1 *	136.28	9.45 *
T_L_ (min)	0.03	3.77 *	0.03	1.1 *
P (cm^2^ h^−1^)	0.50	0.08 *	0.76	0.05 *

F1, F2, F7, and F8. F1: formulation with FA (Ferulic acid) and sunscreens (5% butyl methoxydibenzoylmethane and 10% ethylhexyl methoxycinnamate); F2: formulation with CF (Cicloferulic^®^) and sunscreens (5% butyl methoxydibenzoylmethane and 10% ethylhexyl methoxycinnamate); F7: formulation with FA and no sunscreens; F8: formulation with CF and no sunscreens. J = Steady-state FA flow; T_L_ = lag time; P = Permeability coefficient. (*) This sample exhibited modified Higuchi kinetics.

## Data Availability

The original contributions presented in this study are included in the article. Further inquiries can be directed to the corresponding author.

## Abbreviations

The following abbreviations are used in this manuscript:

FA	Ferulic Acid
CF	Cicloferulic^®^
HPβ-CD	Hydroxypropyl β-cyclodextrin
PVP K30	Polyvinylpyrrolidone K30
FTIR	Fourier Transform Infrared Spectroscopy
DSC	Differential Scanning Calorimetry
TG/DTG	Thermogravimetry / Thermogravimetric Derivative
DR X	X-ray Diffraction
DPPH	1,1-diphenyl-2-picrylhydrazyl
SPF	Sun protection factor
UV	Ultraviolet
ROS	Reactive oxygen species
CD	Cyclodextrin
ATR	Total Attenuated Reflectance
MTT	3-(4,5-dimethylthiazol-2-yl)-2,5-diphenyltetrazolium bromide
DMEM	Dulbecco’s Modified Eagle Medium
OECD	Organization for Economic Cooperation and Development
GLP	Good Laboratory Practice
DCFH-DA	2′,7′-dichlorofluorescein diacetate
UPLC	Ultra Performance Liquid Chromatography
CCDC	Cambridge Crystallographic Data Center
